# Probing the Challenge of P‐Type Semiconductors in Long‐Chain VOC Detection: 3D Micro‐Flower Zinc Cobaltate Heterojunction Sensors

**DOI:** 10.1002/advs.202502646

**Published:** 2025-06-20

**Authors:** Kewei Liu, Zichen Zheng, Yiwen Zhou, Carla Bittencourt, Marc Debliquy, Chao Zhang

**Affiliations:** ^1^ College of Mechanical Engineering Yangzhou University Yangzhou 225127 P. R. China; ^2^ Jiangsu Key Laboratory of Surface Strengthening and Functional Manufacturing Yangzhou University Yangzhou 225127 P. R. China; ^3^ Service de Science des Matériaux Faculté Polytechnique Université de Mons Mons 7000 Belgium; ^4^ Research Institute for Materials Science and Engineering Chimie des Interactions Plasma‐Surface Université de Mons 20 Place du Parc Mons 7000 Belgium

**Keywords:** chemiresistive sensor, heteroatom doping, mildew monitoring, n‐p heterojunction, tetradecane

## Abstract

Tetradecane, a long‐chain alkane recently recognized as a volatile marker for early‐stage mildew detection in stored grains and as a reference material in hydrocarbon studies, presents significant challenges for detection due to its inherent low reactivity and substantial molecular size. This study reports the synthesis of a microflower‐like Fe@WO_3_/ ZnCo_2_O_4_ heterostructure for effective tetradecane sensing. The Fe@WO_3_/ ZnCo_2_O_4_ n‐p junctions demonstrated significant alterations in electrical conductivity upon exposure to tetradecane at room temperature. The sensor achieved a reasonable detection limit of 78.4 ppb and a rapid recovery time of 36 s. The remarkable sensing performance is attributed to the synergistic interactions among multiple heterojunction interfaces, doping‐induced active sites, the presence of oxygen vacancies, high‐energy crystallographic facets, reduced grain size, and enhanced crystallinity, as supported by density functional theory calculations and molecular dynamics simulations. This work identifies a promising candidate for the detection of distinct volatile organic compounds, warranting further exploration in agricultural and emission monitoring applications while addressing a critical gap in metal oxide semiconductor sensors for the detection of large‐molecule gases.

## Graphical abstract

Fe@WO_3_/ZnCo_2_O_4_ n‐p heterojunction sensor exhibits excellent room‐temperature sensing toward tetradecane, with a low detection limit of 78.4 ppb and short recovery time. The good performance is attributed to synergistic effects from heterojunctions, heteroatom doping, oxygen vacancies, high‐energy facets, and theoretical validations.

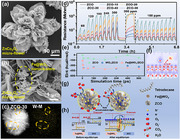

## 1 Introduction

Gas sensors have gained significant interest in recent years due to their potential applications in environmental monitoring, industrial safety, and health diagnostics.^[^ ^1^, ^2^, ^3^, ^4^, ^5^ ^]^ Among various gases, alkanes hold particular importance due to their involvement in numerous industrial processes and their potential environmental impacts. Tetradecane (C_14_H_30_), a representative long‐chain hydrocarbon, is extensively used in lubricants, adhesives, sealants, and surface coatings, and has been identified as a dominant volatile organic compound (VOC) emitted from indoor materials during renovation, with a high indoor/outdoor ratio indicating its potential as a marker of indoor VOC sources.^[^ ^6^ ^]^ Besides, the vapor of n‐tetradecane is heavier than air and may accumulate in low‐lying areas. In the presence of an ignition source such as heat, flame, or spark, leaked vapors can pose a significant fire or explosion hazard.^[^ ^7^ ^]^ Furthermore, tetradecane has been identified among the VOCs linked to microbial metabolism and spoilage in stored grains and food products.^[^ ^8^ ^]^ While not a specific biomarker, it is frequently detected as a secondary metabolite during fungal colonization or mildew development.^[^ ^9^, ^10^ ^]^ Thus, variations in its concentration may serve as an auxiliary indicator of early microbial contamination. Nevertheless, the detection of long‐chain hydrocarbons in gas sensing presents inherent challenges due to their low volatility and weak interactions with sensor surfaces, resulting in reduced adsorption efficiency and attenuated sensor response, especially when compared to smaller, more volatile analytes.

Traditionally, metal oxide semiconductors (MOSs) have been widely studied for gas‐sensing applications because of their high sensitivity, affordability, and simple fabrication process.^[^ ^11^, ^12^ ^]^ Among these materials, zinc cobalt oxide (ZnCo_2_O_4_), a p‐type semiconductor with a spinel structure, has been recognized as a potential candidate owing to its superior thermal stability, diverse valence states, and relatively high intrinsic conductivity arising from the Co^3+^/Co^2+^ redox couple.^[^ ^13^, ^14^, ^15^ ^]^ These properties make ZnCo_2_O_4_ particularly suitable for photodetector and electrochemical applications, such as supercapacitors and electrocatalysis.^[^ ^16^, ^17^, ^18^ ^]^ The synergistic interaction between Zn^2+^ and Co ions enhances its electronic conductivity and structural stability.^[^ ^19^, ^20^ ^]^ However, the practical use of pristine ZnCo_2_O_4_ in gas‐sensing applications has been significantly limited by its inherently low surface reactivity, specifically in terms of weak adsorption and catalytic activation of gas molecules. In contrast, many conventional metal oxide gas sensors suffer from excessively high baseline resistance, which compromises signal stability and increases power consumption. ZnCo_2_O_4_, characterized by its low baseline resistance, offers a potential pathway to develop gas sensors that are both stable and energy‐efficient. Additionally, it provides a valuable reference and design direction for future studies focused on similar challenges in p‐type semiconductor materials.^[^ ^21^ ^]^

The enhanced sensing performance of p–n heterojunctions is mainly attributed to the built‐in electric field at the interface, which facilitates charge separation, suppresses recombination, and improves carrier transport, thereby enhancing sensitivity.^[^ ^22^ ^]^ Moreover, the p–n heterojunction increases the active surface area and reaction interface, providing additional sites for gas adsorption and thereby accelerating response and lowering the detection limit (LoD). By integrating materials with distinct electronic structures and conductivities, the heterojunction enables the synergistic modulation of band alignment and Fermi level position, thereby enhancing charge transfer efficiency and gas‐surface interactions. This modulation also optimizes adsorption‐desorption dynamics, improving both selectivity and sensitivity toward specific target gases. Furthermore, the well‐defined heterojunction interface facilitates efficient charge separation and suppresses carrier recombination, which not only stabilizes the output signal but also minimizes drift and measurement errors during long‐term operation. Through these combined effects, p–n heterojunctions significantly enhance gas sensor performance, enabling rapid and reliable detection of low‐concentration analytes. The enhanced adsorption and charge separation also make them suitable for detecting long‐chain hydrocarbons, offering potential uses in various practical application scenarios.^[^ ^23^, ^24^, ^25^ ^]^

Incorporating iron‐doped tungsten oxide (Fe@WO_3_) into ZnCo_2_O_4_ matrices was reported to lead to significant enhancements in gas sensing performance. N‐type WO_3_, with its high surface area and superior electronic conductivity, improves sensor sensitivity and selectivity.^[^ ^26^ ^]^ Doping the n‐type WO_3_ with Fe ions modifies its electronic properties by increasing the number of active sites for gas adsorption and thereby optimizing the overall sensor response. The substitution of Fe^3+^ for W^6+^ creates oxygen vacancies, promoting gas adsorption and improving charge separation, which collectively increases the sensitivity and efficiency of the gas‐sensing process.^[^ ^27^, ^28^ ^]^

This study proposes an innovative strategy for engineering room‐temperature gas sensors using Fe‐doped WO₃ loaded on ZnCo_2_O_4_ micro‐flowers, resulting in improved tetradecane sensing performance facilitated by the n‐p heterostructure. This configuration achieved a low LoD of 78 ppb, a high response of approximately 22.5 for 20 ppm, and a rapid recovery time (t_rec_) of approximately 36 s for 300 ppm. Density functional theory (DFT) calculations and molecular dynamics (MD) simulations were performed to elucidate the improved sensing mechanism. By leveraging the synergistic effects of the composite materials, this research aims to enhance sensor performance through improved adsorption, charge transfer, and catalytic activity, contributing to the advancement of efficient gas sensing technologies for practical use. Moreover, this study seeks to elucidate the mechanisms underlying gas‐sensing behavior and evaluate the potential of ZnCo_2_O_4_‐based materials for application of crop quality inspection, thereby contributing to the advancement of detection systems for long‐chain alkanes.

## 2 Results and Discussion

### 2.1 Characterization Results

**Figure 1:**
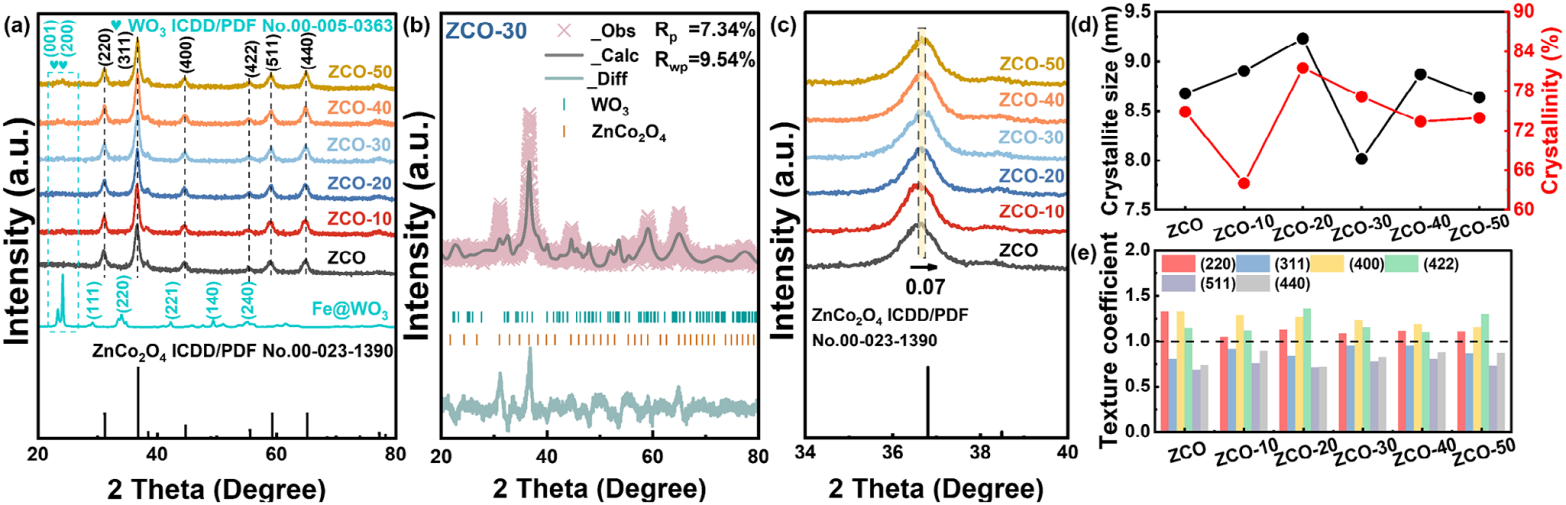
a) Full XRD of Fe@WO_3_, pure ZCO, and ZCO‐(10, 20, 30, 40, and 50). b) Rietveld refinement of the XRD pattern for ZCO‐30. c) Partial enlarged XRD of Fe@WO_3_, pure ZCO, and ZCO‐(10, 20, 30, 40, and 50). d) Calculated grain size and crystallinity. e) Texture coefficient values.

The crystal structure of the synthesized samples was examined via X‐ray diffraction (XRD) using a Bruker D8 Advance diffractometer with a Cu Kα source (λ = 1.5406 Å). The diffraction data were collected over a 2θ range of 20°–80° at a scan rate of 0.02°/min. The resulting XRD patterns were analyzed to identify the phase composition and structural properties of the samples, as displayed in **Figure** **1**. Characteristic peaks were indexed according to the standard reference patterns of pure ZnCo_2_O_4_ (ZCO) from the ICDD database (ICDD#00‐023‐1390), thereby confirming the development of a cubic spinel phase with *Fd‐3m* space group.^[^ ^29^ ^]^ The crystal phase of the composite containing WO_3_ (ICDD#00‐005‐0363) and ZCO was identified in Fe@WO_3_‐loaded ZCO samples, with no additional extraneous peaks detected. The intensities of the (001) and (200) reflections in the Fe@WO_3_ loaded ZCO samples elevated with the rising Fe@WO_3_ loading, indicating the successful synthesis of the heterostructured composites. To meticulously elucidate the crystal structure of ZCO‐30, an exhaustive XRD Rietveld refinement analysis was performed, as illustrated in Figure 1b. This analysis achieved a weighted residual (R_wp_) of 9.54%, signifying excellent concordance with the original XRD data. The findings conclusively affirm that ZCO‐30 crystallizes in a cubic system, corresponding to the *Fd‐3m* space group. Furthermore, the slight redshift (≈0.07°) observed in the XRD pattern was attributed to the diffusion of ^VI^W^6+^ ions (ionic radius = 0.600 Å) into the ZCO lattice, occupying positions ^VI^Co^2+^/^VI^Co^3+^ (0.745/0.610 Å) in accordance with Bragg's Law (Figure 1c). ^30^ With the mismatch between W^6+^ and both cobalt ions remaining below 20%, the relatively lowMismatch indicates a high probability of substitutional doping. This heterovalent doping phenomenon suggests lattice shrinkage and localized stress concentration, effectively tuning the electronic structure and surface activity. The peak positions and intensities were used to evaluate the crystallite size and crystallinity, as evidenced in Figure 1d and Table S1 (Supporting Information). With the increase in loading amount, the crystalline sizes (typical crystal size ≈9 nm) generally increased first from ZCO to ZCO‐20 and then showed a downward trend, attributed to the following phenomenon. In the initial stage, the introduction of Fe@WO_3_ as a nucleation site at the grain boundaries of ZCO contributes to the growth of ZCO grains, facilitating the aggregation and development of the crystals. However, as the amount of Fe@WO_3_ loading continues to increase, Fe@WO_3_ particles may cover the ZCO surface or distribute along the grain boundaries known as the “grain boundary pinning effect”, thus preventing grain movement and halting further grain growth.^[^ ^31^ ^]^ Additionally, the presence of sharp and well‐defined peaks indicates that the synthesized materials exhibit good crystallinity, which varies with the loading amount. The initial introduction of Fe@WO_3_ causes lattice distortion in ZCO, leading to a reduction in crystallinity. However, with increasing loading, interfacial interactions between Fe@WO_3_ and ZCO reach a favorable equilibrium, facilitating the formation of a more ordered interface and thereby improving the overall crystallinity of the composite. However, with further increases in the amount of Fe@WO_3_, the interfacial stress or distortion may increase, potentially weakening the crystal ordering of ZCO once again. The combination of small crystallite size (8.0 nm) and high crystallinity (77.2%) was observed in the ZCO‐30 sample. Furthermore, the texture coefficient (TC) value was calculated to investigate the prioritized orientation, as depicted in Figure 1e and Table S2 (Supporting Information). The loading of Fe@WO_3_ did not cause reorientation, indicating that the (220), (400), and (422) planes experienced preferential growth and gradually became exposed on the surface. The surface energies of different crystal planes were calculated using Equation (1), yielding values of 2.35, 1.79, and 3.60 J m^−2^ for the (220), (400), and (422) planes, respectively.

(1) $$E_{Surf}=\frac{E_{Slab}-nE_{Bulk}}{2A}$$

where *E_Slab_* and *E_Bulk_* represent the total energies of the slab and bulk unit cells, respectively. A represents the surface area of the crystal plane per repeating unit in the slab model. The (422) facet, identified as a high‐index surface with a calculated high surface energy, suggests a higher density of undercoordinated atoms and enhanced surface reactivity, which facilitates the adsorption of bulky, low‐volatility long‐chain VOCs such as tetradecane.^[^ ^32^ ^]^ The optimized surface structures are shown in Figure S3 (Supporting Information). Notably, an upward trend appeared in the standard deviation (σ) and ZCO‐30 obtained the highest value (Table S2, Supporting Information), signifying that heterogenous nucleation favored the formation of new phases or reduced the energy barrier for nucleation at structural imperfection in ZCO, thereby enhancing the generation of small grains, which corresponds with the results show in Figure 1d.^[^ ^33^ ^]^

**Figure 2:**
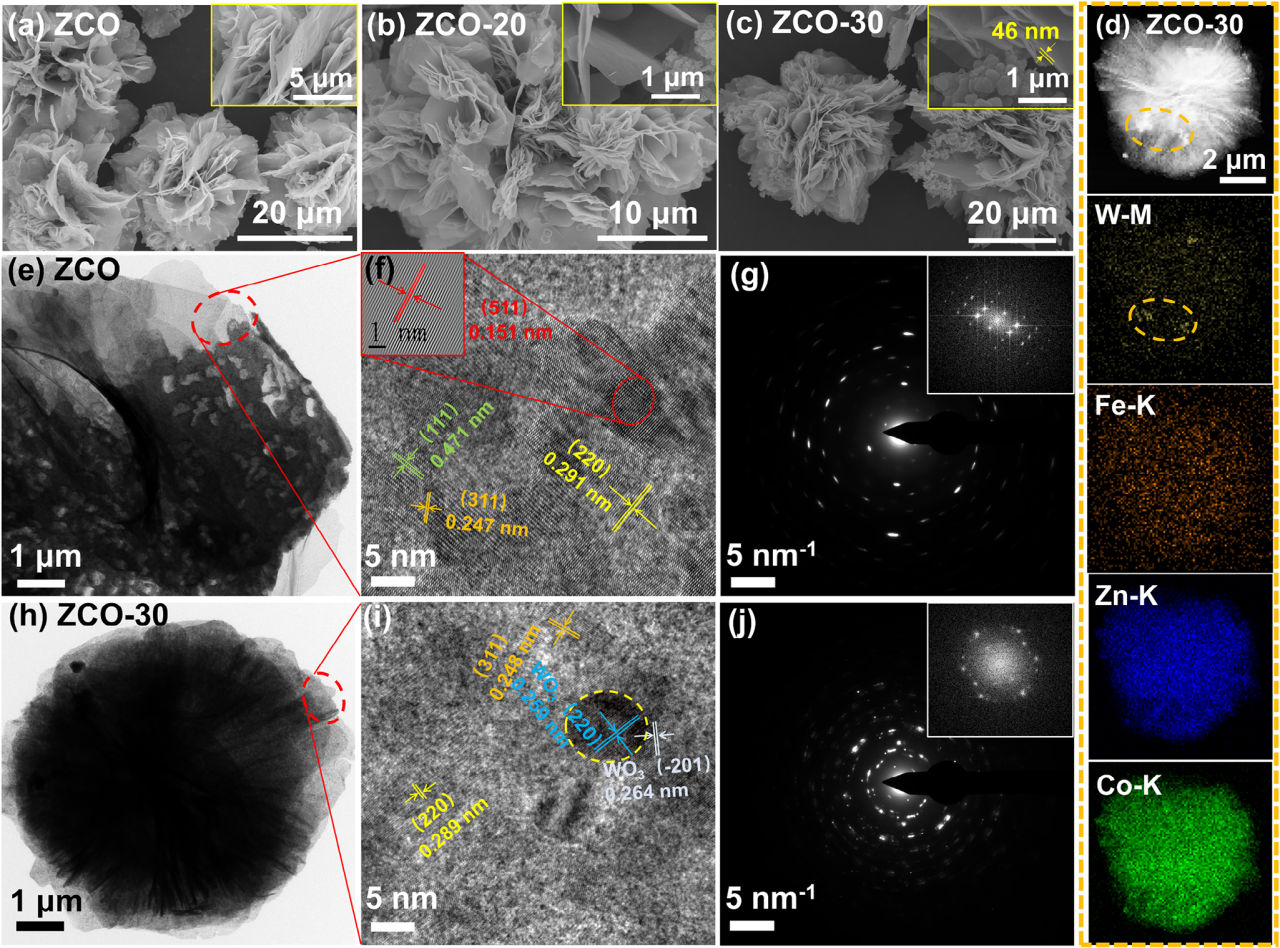
Structural characterization of pristine ZCO and the composites. FE‐SEM images of a) ZCO, b) ZCO‐20, and c) ZCO‐30. Inset: the magnified SEM images of them. d) EDX elemental mapping of ZCO‐30. TEM e, h) and high‐resolution TEM images f, i) of ZCO and ZCO‐30. The crystallites in the images are color‐coded depending on their lattice fringes. SAED analysis of g) ZCO and j) ZCO‐30 by drop‐casting suspension on TEM grids and drying in air. Inset: FFT profiles.

To examine the surface features of the synthesized samples, Scanning Electron Microscopy (SEM) and Transmission Electron Microscopy (TEM) were employed, as illustrated in **Figure** **2**.

The pristine ZCO exhibits a flower‐like microstructure composed of curved nanosheet‐shaped petals. The dimensions of the micro‐flower typically range from 20 to 30 µm, with petal thicknesses of approximately 40 ± 10 nm. A porous architecture is evident on the 2D nanosheets, which arises from the release of NO_2_ during the thermal decomposition. The complex structure provides a pathway for electron transport. Besides, the wide interlayer gaps between the ultrathin nanosheet petals allow easier penetration and diffusion of large gas molecules. These inter‐sheet gaps may induce transient localized increases in gas concentration upon exposure, effectively serving as natural enrichment cavities that enhance the probability of gas‐surface interactions, contributing to improved sensitivity toward long‐chain hydrocarbons.^[^ ^34^ ^]^ Moreover, the flower‐like ZCO microstructure is preserved alongside the development of clustered Fe@WO_3_, which consists of irregular nanoparticles anchored to the ZCO surface and extending outward during the solvothermal reaction, resulting in a decorative heterostructure as shown in Figure 2a‐c and Figure S4 (Supporting Information). The proportion of nanoclusters surrounding the ZCO nanosheets significantly increased with the rising loading amount of Fe@WO_3_. The homogenous distribution of W, Fe, Zn, and Co atoms on the surface of the ZCO‐30 micro‐composite is further confirmed by EDX mapping in Figure 2d. Notably, the W element is predominantly located on the nanoclusters on the ZCO surface (indicated by the orange circle), illustrating the formation of heterogeneous interfaces that enhance interfacial charge transfer capability. Besides, the EDX spectra in Figure S5 (Supporting Information) provided elemental composition data of pristine ZCO and ZCO‐30 samples. Characteristic peaks of W and Fe demonstrated the successful synthesis of the composite structure, which further confirmed distinct phase formation in the Fe@WO_3_/ZnCo_2_O_4_ heterojunction. TEM analysis offered key insights into the crystal structure and interfaces of both pristine ZCO and ZCO‐30. Figure 2f,i display lattice patterns at the edges of the petals shown in Figure 2e,h (highlighted by the red circle), with distinctly interplanar spacing, consistent with well‐defined diffraction spots and rings in Selected Area Electron Diffraction (SAED) images (Figure 2g,j). These observations indicate that high crystallinity is maintained both prior to and following the formation of the heterojunction. The measured lattice fringes in Figure 2f are 0.151, 0.247, 0.291, and 0.471 nm, associated with the typical ZCO planes of (511), (311), (220), and (111), respectively, aligning with the XRD results (Figure 1a). As illustrated in Figure 2i, the lattice fringes in the vicinity of the dark area (highlighted by a yellow oval) exhibit interplanar spacings of 0.259 and 0.264 nm, which correspond to the (220) and (‐201) planes of WO_3_ respectively, indicating the coexistence of ZCO and WO_3_. Interestingly, while pristine ZCO displays characteristics of a single crystal, as evidenced by SAED and fast Fourier transform (FFT) profiles, the ZCO‐30 sample reveals a typical polycrystalline feature characterized by a series of concentric rings with prominent bright spots (Figure 2g,j). This transformation is likely attributed to the influence of Fe@WO_3_ on the growth dynamics of ZCO crystals. The introduction of WO_3_ initiates nucleation at different points on the ZCO surface, promoting the formation of new nuclei and facilitating growth in multiple directions rather than along a single orientation. Additionally, the small particle of Fe@WO_3_ possesses high surface energy, leading to the clustering and rearrangement of grains, thereby favoring the emergence of a polycrystalline structure.^[^ ^35^ ^]^ Consequently, heterostructured Fe@WO_3_‐loaded ZCO composites are effectively synthesized.

**Figure 3:**
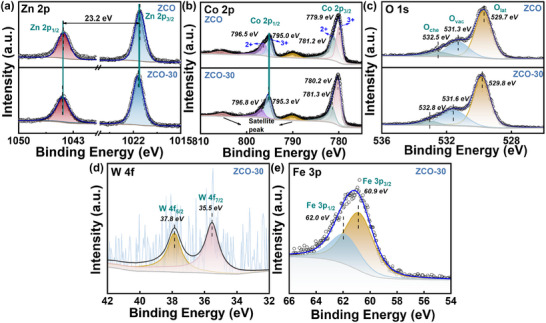
XPS deconvoluted core‐level spectra of ZCO and ZCO‐30 of a) Zn 2p, b) Co 2p, c) O 1s, d) W 4f, and e) Fe 3p.

The elemental composition and electronic structure of pristine ZCO and Fe@WO_3_‐loaded ZCO were investigated using X‐ray Photoelectron Spectroscopy (XPS). As shown in **Figure** **3**a, the Zn 2p peaks of ZCO are located at 1021.1 and 1044.3 eV, corresponding to the Zn 2p3/2 and Zn 2p1/2 peaks, respectively. The binding energy separation between these peaks is estimated as 23.2 eV, indicating that Zn exists in its typical oxidation state of Zn^2+^.^[^ ^36^, ^37^ ^]^ The Zn 2p peaks of ZCO‐30 have similar binding energies as the pristine ZCO. The Co 2p spectrum reveals two distinct peaks characterized by broad and asymmetric profiles, attributed to Co 2p1/2 and Co 2p3/2, along with two low‐intensity satellites (Figure 3b).^[^ ^38^ ^]^ The spin‐orbit doublet corresponding to Co 2p3/2 and Co 2p1/2 was further deconvoluted into components at 780.2 and 795.3 eV associated with Co^3+^ and at 781.3 and 796.8 eV corresponding to Co^2+^. This suggests the coexistence of Co^3+^ and Co^2+^ in ZCO. The amount of Co ions with different oxidation states following the loading of Fe@WO_3_ attributed to changes in the local electronic environment. Moreover, the existence of Co^2+^ is linked to the generation of oxygen vacancies (O_vac_), which serve as additional active sites, thereby enhancing gas sensing performance.^[^ ^39^ ^]^ The existence of the satellite peaks at binding energies of 789.9 and 805.7 eV further corroborates the presence of cobalt oxides. Additionally, the O 1s spectrum, as depicted in Figure 3c, is deconvoluted into three components aligned with lattice oxygen (O_lat_) (≈529.7 eV), O_vac_ (≈531.3 eV), and chemisorbed oxygen species (O_che_) (≈532.5 eV).^[^ ^40^ ^]^ A significant increase in the area ratios of (O_vac_+O_che_) is observed following the loading of Fe@WO_3_, indicating changes in surface oxygen states.^[^ ^41^ ^]^ Additionally, the observable shift of XPS peaks to higher binding energies for ZCO after Fe@WO_3_ loading is primarily due to electron withdrawal from ZCO to WO_3_ upon interaction, resulting in a decrease in electron density around the Zn and Co atoms, thereby increasing the binding energy (Figure 3a–c). This is also evidenced by the increased content of Co^3+^ in ZCO‐30 compared to pristine ZCO, as shown in Figure 3b. The Co^3+^ ratios in ZCO and ZCO‐30 are calculated to be 46.7% and 67.3%, respectively. The increased Co^3+^ content in ZCO‐30 contributes to a higher hole concentration, thereby enhancing the intrinsic p‐type conductivity and facilitating improved charge transport. The doublet W 4f has peaks at 37.8 and 35.5 eV, corresponding to the binding energies of the W4f5/2 and W4f7/2 orbitals in the W^6+^ oxidation state, respectively, confirming that the loaded nanoclusters consist of stoichiometric WO_3_ (Figure 3d).^[^ ^42^ ^]^ Furthermore, as shown in Figure 3e, the doublet Fe 3p peaks are positioned at 62.0 eV (Fe 3p1/2) and 60.9 eV (Fe 3p3/2), respectively, indicating the presence of Fe^3+^ in the Fe@WO_3_ composite.^[^ ^43^ ^]^

**Figure 4:**
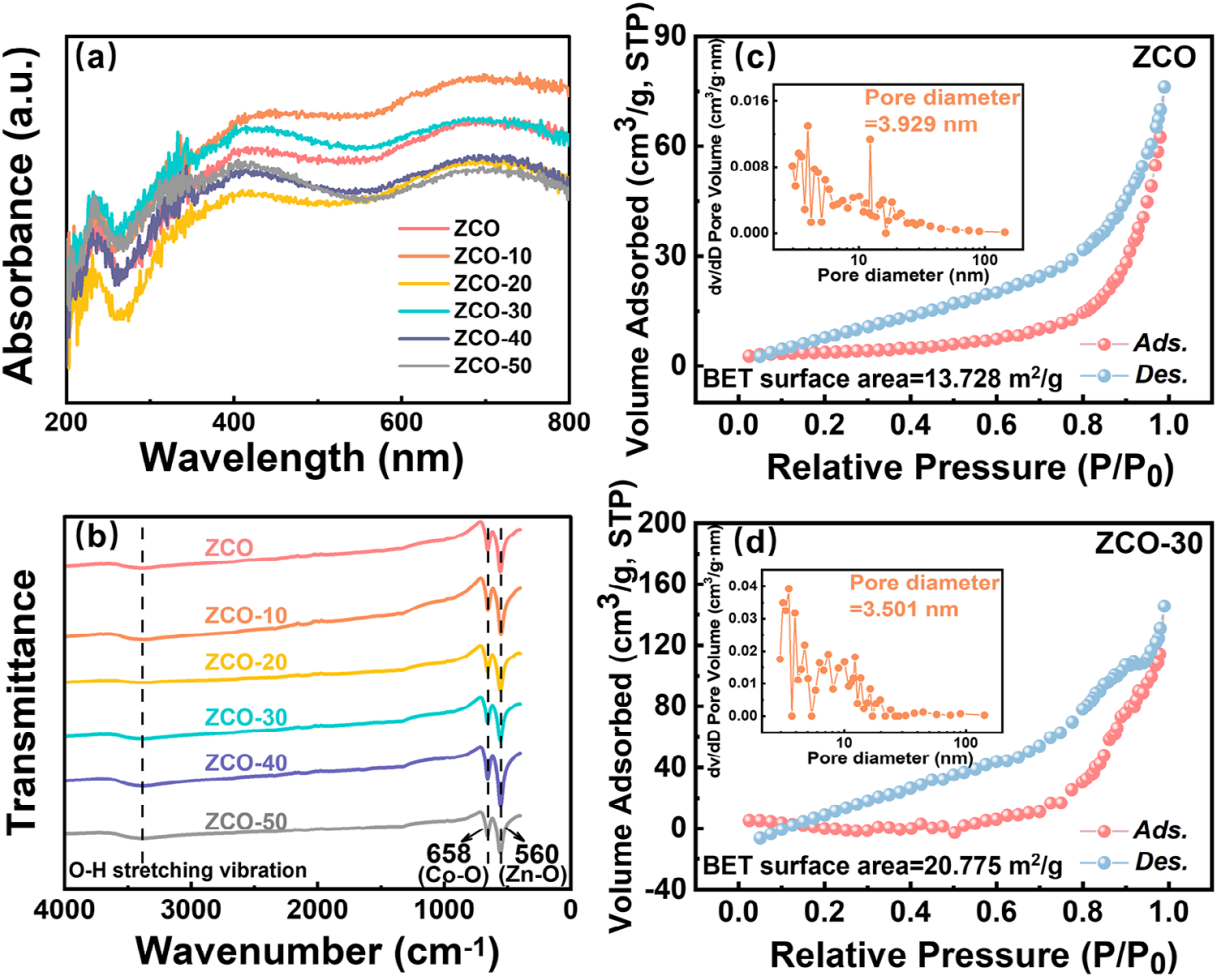
a) UV–vis DRS spectrum and b) FTIR spectra of all samples. Nitrogen adsorption‐desorption isotherms. Inset: Barrett‐Joyner‐Halenda pore‐size distributions of c) ZCO and d) ZCO‐30 through fitting the desorption branch of isotherms.

Ultraviolet‐visible Diffuse Reflectance Spectra (UV–vis DRS) was used to investigate the band structure (**Figure** **4**a). All samples exhibited broad absorption across the 200–800 nm range. However, direct bandgap determination from the Kubelka‐Munk‐transformed spectra [F(R)] was hindered by near‐zero reflectance. This limitation arises because conventional UV–vis transmission methods are invalid for such materials due to signal saturation at near‐zero transmittance.^[^ ^44^ ^]^

Figure 4b presents the FTIR analysis of both pristine ZCO and Fe@WO_3_‐loaded ZCO, revealing two prominent sharp peaks within the range of 560 to 660 cm^−1^. These peaks correspond to the strong metal‐oxygen bonds characteristic of the spinel structure. The peak at 560 cm^−1^ is associated with the Zn^2+^ ions in tetrahedral coordination, while the peak at 658 cm^−1^ stems from the Co^3+^ ions in octahedral coordination. These spectral features reflect the F1u vibrational modes indicative of the metal‐oxygen bonding in the spinel structure of ZCO.^[^ ^45^ ^]^ Additionally, the peak at around3500 cm^−1^ corresponds to the stretching vibrations of O‐H bonds, suggesting the presence of water molecules or surface‐adsorbed moisture within the samples.^[^ ^46^ ^]^ Furthermore, the surface specific areas (SSA) of pristine ZCO and ZCO‐30 were measured at 13.73 and 20.78 m^2^ g^−1^, respectively, with average pore diameters of 3.93 and 3.50 nm, and total pore volumes of 0.12 and 0.26 cm^3^ g^−1^ as shown in Figure 4c,d. Both samples exhibit mesoporous size distributions. The incorporation of Fe@WO_3_ increased the SSA compared to pristine ZCO, and the enhanced total pore volume of ZCO‐30 facilitated VOC diffusion, thereby improving the sensing performance.^[^ ^41^ ^]^

### 2.2 Gas Sensing Performance

**Figure 5:**
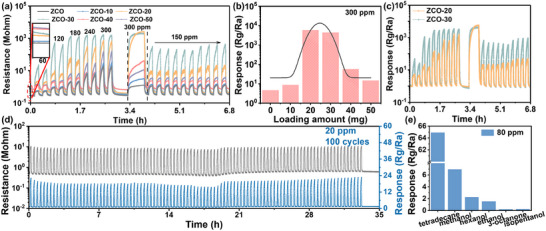
Tetradecane gas performance of pristine ZCO and composites‐based sensors. All measurements are performed at room temperature. a) Dynamic resistance curves of all samples to 60–300 ppm tetradecane. b) The responses of all samples for 300 ppm tetradecane at different loading amounts. c) Response‐recovery curves of ZCO‐20 and ZCO‐30 to 60–300 ppm tetradecane. d) Repeatable characteristic of ZCO‐30 to 20 ppm tetradecane in 100 consecutive responses. e) Selectivity to 80 ppm tetradecane and other interfering gases of ZCO‐30.

The gas sensing properties of pristine ZCO and Fe@WO_3_ loaded composites were systematically evaluated using a custom‐designed testing system, as illustrated in Figure S2. The dynamic response/recovery curves of all tested samples in response to tetradecane concentrations ranging from 60–300 ppm at room temperature are presented in **Figure** **5**a. These curves exhibit predominant p‐type characteristics, evidenced by a marked increase in resistance when exposed to the reducing gas tetradecane. Notably, the reference resistances of the gas sensors demonstrated an upward trend with increased loading amounts, which can be attributed to the establishment of n‐p heterojunction, resulting in carrier depletion at the interface due to enhanced electron transfer from the n‐type Fe@WO_3_ to the p‐type ZCO, where the primary charge carriers are holes, thereby reducing the overall carrier density.^[^ ^47^ ^]^ Analysis of the dynamic resistance curves across varying tetradecane concentrations revealed that the sensing performance of the composites significantly surpassed that of pristine ZCO, achieving a response of ov7.5 at 60 ppm of the target gas. Among the studied composites, the ZCO‐30 sensor exhibited the highest sensitivity to tetradecane, which is attributed to the optimal synergistic interactions between Fe@WO_3_ and ZCO. The heterovalent Fe^3+^ doping introduced lattice distortion and local electronic perturbations, generating surface defects and interfacial stress that facilitated charge transport and enhanced the heterojunction coupling, contributing to yielding a response 6.8 times greater than that of pristine ZCO (Figure 5c). A decrease in sensitivity was observed when the Fe@WO_3_ loading content exceeded 30 mg, primarily because of the saturation of active sites on the surface and modifications in the electronic band structure, which hinders the charge transfer processes, and diminish the interaction capacity between tetradecane and the sensing layer. Additionally, excessive Fe@WO_3_ may form a physical barrier that limits gas diffusion to the active interface, while also introducing redundant heterojunctions and excessive lattice distortion, both of which disrupt carrier transport pathways and promote recombination. This phenomenon results in a reduction of the sensor's signal alongside an increase in reference resistance, thereby indicating that 30 mg is the optimal Fe@WO_3_ loading for gas‐sensing applications.

The rate of sensitivity variation of ZCO‐30 in the higher concentration range (180–300 ppm) (648%/ppm) was significantly lower than that recorded in the 60–180 ppm range (2240%/ppm). This decrease in sensitivity at elevated concentrations can be attributed to charge‐release saturation, which occurs as the finite number of available active surface sites becomes fully occupied with increasing analyte concentrations.^[^ ^48^ ^]^ The response values exhibited a volcano‐shaped trend, as determined through GaussAmp fitting, with the peak response occurring around 25 g at 300 ppm tetradecane. However, as depicted in Figure 5c, it is calculated that the t_res_ time to reach the steady stated for ZCO‐20 is 1.3 times that of the ZCO‐30, with the t_rec_ for ZCO‐20 and ZCO‐30 being 0.84 min and 0.60 min, respectively. This indicates that although the ZCO‐20 sample achieves a higher response, it requires more time for both stabilization and recovery. Furthermore, ZCO‐20 sensor exhibits a strong linear correlation between response and tetradecane concentration, indicating its potential for quantitative analysis. The response versus concentration fitting lines for ZCO‐20 are illustrated in Figure S6 (Supporting Information), revealing a theoretical LoD of 78.4 ppb, determined using the formula LoD = 3 × (Standard Deviation/Slope) based on 100 baseline resistance measurements. Additionally, all sensors exhibited stable dynamic resistance and response curves with minimal fluctuations, a characteristic most evident in ZCO‐30. In comparison to pristine ZCO, the ZCO‐30 sensor demonstrated significant improvements in resistance and response curves over 10 repeated cycles of detecting 150 ppm tetradecane, highlighting the enhancement in repeatability afforded by the incorporation of Fe@WO_3_ in ZCO‐based gas sensors (Figure 5a). Moreover, the ZCO‐30 composite displayed not only excellent dynamic sensing performance for high concentrations of tetradecane (60–300 ppm) but also exhibited good response and recovery at a lower concentration of 20 ppm, achieving a response of 22.5, along with stable baseline performance over 100 cycles, suggesting reliable real‐time repeatability (Figure 5d). The state‐of‐the‐art tetradecane gas sensors are given in **Table** **1**. Furthermore, temperature‐dependent dynamic response curves of the sensor are presented in Figure S7 (Supporting Information), illustrating its behavior under varying thermal conditions. As the operating temperature increases from room temperature to 200 °C, the ZCO‐20 composite shows a clear trend of increasing response, reaching a maximum at 100 °C, followed by a decline. At 100 °C, the sensor exhibits enhanced responses of approximately 3.5, 3.6, and 2.0 times at 60, 120, and 180 ppm tetradecane, respectively. This improvement is attributed to moderate heating, which promotes the volatilization and diffusion of long‐chain hydrocarbons, thereby enhancing gas‐solid interactions and surface reaction kinetics. Notably, the improved performance at 100 °C suggests strong application potential, particularly in scenarios similar to grain storage, where ambient temperatures typically remain below this level.^[^ ^49^ ^]^ In contrast, a marked decrease in response is observed at 200 °C, primarily due to accelerated desorption and reduced physisorption stability of tetradecane, which shortens molecular residence time and weakens gas‐surface interactions.^[^ ^50^ ^]^ The effect is more pronounced in p‐type‐dominated systems, where elevated temperatures increase hole concentration and diminish the heterojunction's ability to modulate charge transport. In Figure 5e, VOCs commonly produced during the mildew process of unhusked rice were selected as detection targets. ZCO‐30 demonstrated the highest sensitivity to 80 ppm tetradecane, with a response that was over 9.3 times greater than its responses to other tested gases. This phenomenon is attributed to that methanol, ethanol, isopentanol, and hexanol are classified as short‐ to medium‐chain VOCs, characterized by relatively small molecular sizes, higher polarity, and faster desorption rates, which result in weaker and less stable sensor responses with elevated highest occupied molecular orbital (HOMO) as shown in Figure S8 and Table S3 (Supporting Information). Besides, short‐chain VOCs typically adsorb as isolated molecules. In contrast, long‐chain VOCs tend to exhibit cooperative adsorption or layer‐by‐layer accumulation due to their extended molecular structure, particularly on porous or high‐surface‐area materials.

**Table 1:** The state‐of‐the‐art tetradecane gas sensors.

Sensor Type	Sensing Materials	Con. [ppm]	Response	t_res_ [s]	Operating Temp. [°C]	LoD [ppm]	Refs.
**Acoustic Sensor**	Non‐receptor‐based	20	≈1 mV	/	RT	<10	[51]
**Piezoresistive Microcantilever Sensor**	poly(ethylene imine)	9.33	250–300 nm	/	RT	0.31	[52]
**Thickness Shear Mode Resonator**	polyethylene	0.014	39 Hz	>100	50	/	[53]
**Chemiresistive Sensor**	WS₂	100	1.1%^b)^	/	RT	/	[54]
	PPy‐functionalized graphene	20	≈2.5%^b)^	/	RT	≈5	[55]
	Fe@WO_3_/ZnCo_2_O_4_	20	22.5^a)^	36	RT	0.008	**This work**

^a)^ represents Rg/Ra;

^b)^ represents (△R/Ra)*100%, and RT is room temperature.

### 2.3 Gas Sensing Mechanism

**Figure 6:**
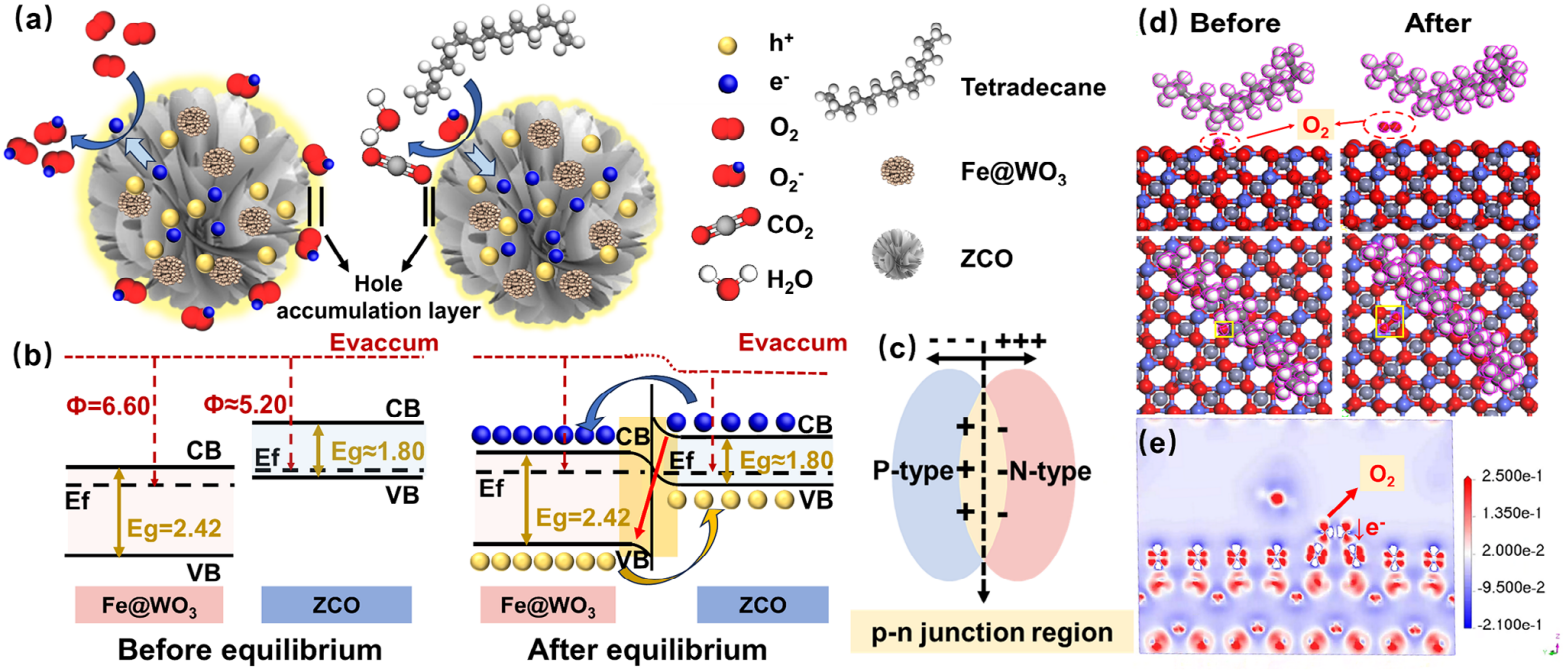
The sensing mechanism of Fe@WO_3_ loaded ZCO sensor. a) Schematic illustration of the gas‐sensing mechanism of Fe@WO_3_ loaded ZCO sensor for detecting tetradecane. b) Energy band structure of Fe@WO_3_ loaded ZCO sensor. c) Situation after the formation of the depletion region. d) The configuration of molecular tetradecane adsorption on ZCO (100) surface with pre‐adsorbed oxygen (top and side view). e) Electron density difference analysis of ZCO with the adsorption of tetradecane and pre‐adsorbed oxygen.

The sensing mechanism of semiconducting metal oxide is predominantly characterized by variations in resistance upon interaction with target gases. **Figure** **6** illustrates the sensing enhancement mechanism of the heterostructured ZCO‐based sensors for the detection of tetradecane gas. The ZCO and Fe@WO_3_ composite materials investigated in this study exhibit sensing behaviors consistent with p‐type MOSs characteristics, implying that holes serve as the primary charge carriers in these sensors. As shown in Figure 6a, under ambient conditions, oxygen molecules from the atmosphere absorb onto the surface of ZCO, leading to the generation of chemisorbed oxygen ions ($O_{2(\mathrm{ads})}^{-}$ used in this work for gas detection at room temperature) through electron removal from the conduction band (Equation (2)). This process generates a Hole Accumulation Layer (HAL) at the surface, facilitating charge transport predominantly along the interfacial region adjacent to the HAL (Figure 6a left). The reduction in electron density corresponds to an increase in hole concentration, which consequently leads to a decrease in the sensor's resistance. Therefore, the baseline resistance is generally low across all sensor configurations. Upon exposure to tetradecane vapor, the interaction between the tetradecane molecules and $O_{2(\mathrm{ads})}^{-}$, promotes electron donation from the reducing gas, which reacts with $O_{2(\mathrm{ads})}^{-}$ and releases electrons back into the semiconductor (Equation (3)). Consequently, the HAL diminishes, resulting in an overall increase in resistance (Figure 6a, right). As the content of tetradecane vapor rises, the thickness of the HALs progressively decreases, enhancing the sensor response. Following exposure to ambient air, the desorption of vapor molecules from the surface of the ZCO‐based sensor allows its recovery to the original state.

(2) $$O_{2\left(\mathrm{ads}\right)}+e^{-}↔O_{2\left(\mathrm{ads}\right)}^{-}\left(T<100\;C\right)$$

(3) $$R-CH_{2}+O_{2\left(\mathrm{ads}\right)}^{-}\to R-C=O+H_{2}O+2e^{-}$$

Moreover, the heterojunction formed between ZCO and Fe@WO_3_ significantly enhances sensing performance. In the context of chemical sensitization, n‐type WO_3_ loading enhances the adsorption of reactive oxygen, thereby promoting the sensing reactions with tetradecane.^[^ ^56^ ^]^ Additionally, electronic sensitization also contributes to improved sensing performance. Owing to the variation in work functions, which are5.2 eV for ZCO and 6.6 eV for Fe@WO_3_, electrons flow from ZCO to Fe@WO_3_, establishing charge equilibrium within the staggered gap.^[^ ^57^, ^58^ ^]^ This movement of electrons is counterbalanced by the migration of holes in the opposite direction, resulting in a hole depletion layer on the ZCO side.^[^ ^59^ ^]^ The reduction in hole concentration is primarily caused by the migration of electrons from the p‐type material, disrupting the internal hole equilibrium and resulting in a reduction in the hole concentration, as illustrated in Figure 6b. Although holes migrate from Fe@WO_3_ to ZCO, the influx of electrons into Fe@WO_3_ locally depletes ZCO of free holes in regions surrounding the Fe@WO_3_ nanoclusters, which increases the reference resistance as the loading amount increases (red arrow). However, this effect does not significantly impact the bulk properties of p‐type ZCO. As illustrated in Figure 6c, the reversed built‐in electric field at the n‐p heterojunction interface promotes efficient charge separation during interactions with gas molecules. This phenomenon results in the displacement of free electrons in WO_3_ away from the junction, thereby enlarging the depletion region near the interface and raising the energy barrier. Consequently, even minor fluctuations in electron density, attributable to gas interactions (specifically surface oxygen concentration), can induce significant changes in resistance. The accumulation of electrons on the WO_3_ side enhances the effect of adsorbed oxygen species, which readily capture electrons from the conduction band of WO_3_, resulting in the formation of oxygen ions. However, this process leads to an overall increase in resistance, as the majority carriers, free holes in p‐type ZCO, are diminished due to the neutralization of reintroduced electrons, thereby decreasing the material's conductivity. The built‐in electric field not only facilitates charge separation but also reduces recombination, thereby amplifying the observed resistance change.

Furthermore, the enhanced sensing performance for tetradecane is correlated with the substitution of Fe^3+^ for W^6+^. Fe doping alters the lattice and charge balance, facilitating the release of terminal oxygen from the WO_3_ lattice, in accordance with Kröger‐Vink notation (Equations (4) and (5)), resulting in the formation of O_vac_, as corroborated by XPS results (Figure 3c).^[^ ^60^ ^]^

(4) $$WO_{3}+Fe_{2}O_{3}\to 2F{e^{\prime \prime \prime }}_{w}+3V_{O}^{\cdot \cdot }+6O_{O}^{x}+W_{W}^{x}$$

(5) $$3/2\;O_{2}+3V_{O}^{\cdot \cdot }\to 3O_{O}^{x}+6h^{\cdot }$$

The surplus of free electrons resulting from the removal of oxygen atoms contributes to an increased electron density, thereby creating numerous active sites for the adsorption of oxygen species (particularly O_che_) and leading to more pronounced changes in electrical conductivity. Additionally, the enhanced disparity in charge carrier concentrations across the n‐p interface intensifies the electric field at the heterojunction, facilitating efficient electron‐hole separation and subsequently improving overall sensing performance.

The sensing mechanism is further elucidated through the characterization of samples. As shown in Figure 2i, the lattice fringes of WO_3_ and ZCO exhibit evident staggering, with a distinct interface between the two phases. This observation highlights the intense contact and effective heterojunction formation that enhances sensing capabilities. Moreover, the microflower architecture formed by stacked nanosheets and nanoparticle assemblies significantly increases the specific surface area (Figure 3c,d). This structural configuration provides multiple pathways for the transmission and diffusion of gas molecules, thereby providing additional active sites for adsorption and facilitating redox reactions with tetradecane gas.

To further validate the proposed mechanism for enhancing sensing material performance, calculations based on DFT were employed. The surface work function of the ZCO (100) plane was calculated using CASTEP to be 4.007 eV, indicating a preferential electron transfer from WO_3_ to ZCO (Figure S9a, Supporting Information). Furthermore, the energy band structure of ZCO, as illustrated in Figure S9b (Supporting Information), reveals a band gap of 0.838 eV. Following the surface cleavage of optimized ZCO crystals, the adsorption energy (E_ads_) of tetradecane molecules on the ZCO (100) surface with pre‐adsorped O_2_ can be determined using Equation (6):

(6) $$E=E_{\mathrm{total}}-\left(E_{\mathrm{surface}}+E_{\mathrm{gas}}\right)$$

where E_total_ represents the total energy of the system in which molecules are adsorbed onto (100) crystal facets of the ZCO surface. E_surface_ denotes the energy of the (100) facet of the ZCO surface. E_gas_ corresponds to the energy of the isolated tetradecane molecules. The calculated E_ads_ of the geometrically optimized adsorption model in Figure 6d is −6.06 eV, of which the negative values imply that the adsorption processes are both spontaneous and exothermic. The notably high E_ads_ suggests a strong interaction between tetradecane molecules and the active sensing surface, indicating a favorable adsorption affinity toward long‐chain VOCs. This interaction is essential for compensating for the low volatility and slow diffusion characteristics of such analytes, thereby facilitating effective detection. It can be observed that the increased distance between the pre‐adsorbed O_2_ molecule and the ZCO surface after geometric optimization reflects the interaction of tetradecane molecules, which release electrons from the chemically adsorbed $O_{2}^{-}$ back to the sensing material, accompanied by the departure of O_2_ molecules. This process facilitates charge transfer or electronic coupling between them. Additionally, the electron density difference in Figure 6e verifies that tetradecane acts as an electron donor, facilitating charge transfer from the $O_{2}^{-}$ and tetradecane gas molecules to the material's surface (red region), which enhances the sensor's electronic response and contributes to improved detection sensitivity.

**Figure 7:**
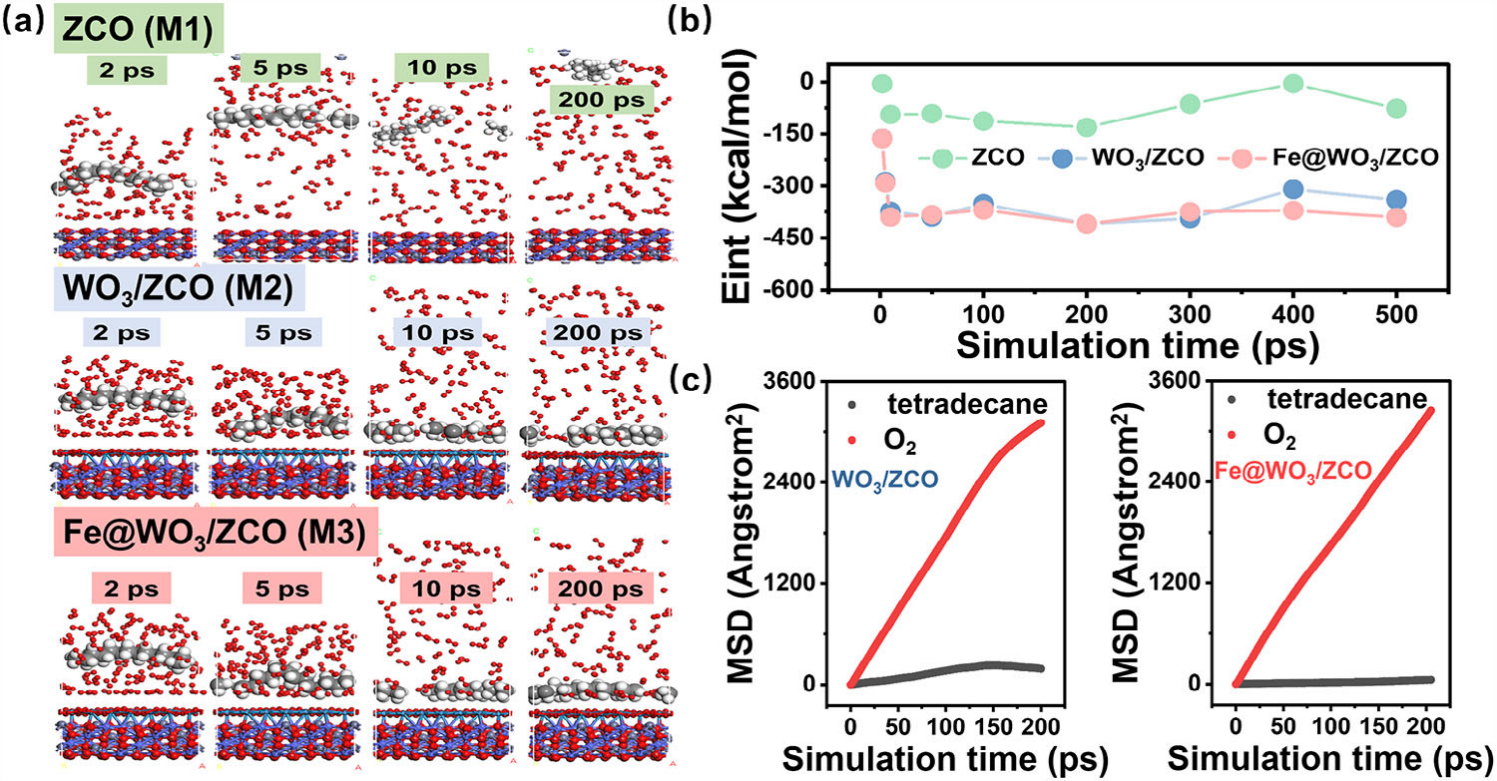
a) MD simulation snapshots, b) E_int_ for the dynamic process of tetradecane and oxygen diffusion on ZCO, WO_3_/ZCO and Fe@WO_3_/ZCO. c) MSD of tetradecane and oxygen diffusion on WO_3_/ZCO and Fe@WO_3_/ZCO.

To further analyze the underlying mechanism, the adsorption and diffusion behavior of tetradecane and oxygen molecules on the heterostructure surface was investigated using MD simulation (conducted at 298 K). The adsorption configuration is provided in Figure S10 (Supporting Information). As seen in **Figure** **7**a, the approach of tetradecane toward the surface and the concurrent dispersion of O_2_ molecules signify a shift in adsorption dynamics. This transition is likely driven by the electron donation from tetradecane, which neutralizes adsorbed oxygen species and promotes the desorption of O_2_. As shown in Figure 7b, a significant reduction in interaction energy (E_int_) is identified between 2 and 5 ps, marking tetradecane's transition into an attractive interaction domain as it approaches the surface. This phenomenon, governed by van der Waals interactions, is a consequence of tetradecane's nonpolar molecular nature. The adsorption dynamics of tetradecane on a pure ZCO‐based model (M1) exhibit distinct fluctuations and instability. Between 10 and 200 ps, the Eint decreases, reaching a minimum of −131 kcal mol^−1^ at 200 ps, indicating the formation of a strong but transient adsorption complex. After 200 ps, significant fluctuations occur, indicating structural rearrangements or partial desorption processes. For the WO_3_/ZCO‐based model (M2) and Fe@WO_3_/ZCO‐based model (M3, Atomic ratio of Fe to W is 2.08%), between 5–50 ps, E_int_ stabilizes at approximately 389 kcal mol^−1^, denoting the formation of a stable adsorption complex. This robust interaction indicates strong gas binding, which could significantly enhance the material's sensing capabilities. The energy fluctuations observed during this period are likely attributable to minor structural realignments or competing adsorption dynamics on the surface. Notably, the E_int_ of M3 is more negative and decreases more sharply between 1 and 10 ps, indicating stronger and faster adsorption of tetradecane and more effective interaction with oxygen molecules compared to the M2. Besides, M3 has lower fluctuations in E_int_ (≈20 kcal mol^−1^) than M2 (>30 kcal mol^−1^), suggesting more stable adsorption complexes. The incorporation of Fe dopants introduces active sites, enhancing interactions with oxygen and hydrocarbons while facilitating electron transfer. Consequently, M3 with Fe doping demonstrates good adsorption strength, stability, and sensitivity. The mean square displacement (MSD) was calculated to quantitatively analyze the interaction between the gas phase and sensing materials (Figure 7c). O_2_ molecules in M2 and M3 adsorption configurations present a high diffusion coefficient (D) of 2.29 × 10^−4^ and 2.55 × 10^−4^ cm^2^ s^−1^, respectively. At the same time, the D value for tetradecane is significantly lower at 2.78 × 10^−5^ for M2 and 5.30 × 10^−6^ cm^2^ s^−1^ for M3 because of adsorption constraints, consistent with the more negative E_int_ and reduced fluctuations observed in the M3 configuration. Furthermore, the better linearity of MSD in M3 implies greater stability in adsorption and desorption dynamics, highlighting the role of Fe doping in optimizing surface interactions and stabilizing the sensing mechanism.

### 2.4 Rice Mildew Detection

The practical applicability of the proposed sensors was evaluated by monitoring VOC emissions from unhusked rice that had undergone an initial 3‐month storage period in a granary. Previous studies have indicated that the quality of freshly harvested rice tends to decline after more than three months of storage, during which the level of tetradecane gradually increases over time.^[^ ^49^ ^]^ As shown in Figure S11 (Supporting Information), the Gas Chromatography‐Mass Spectrometry (GC‐MS) analysis of VOCs emitted from unhusked rice stored for 30 to 100 days reveals the appearance of a detectable tetradecane peak after 30 days, which becomes progressively more pronounced with longer storage durations.

**Figure 8:**
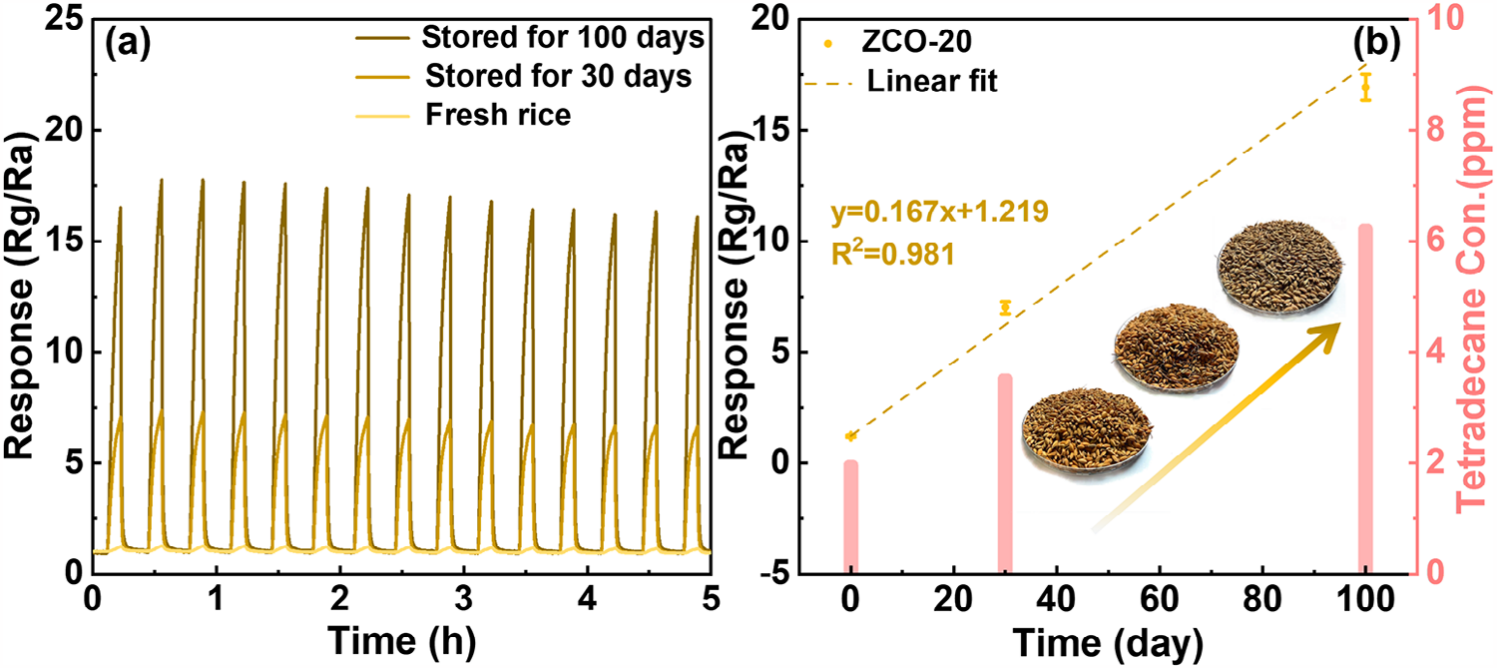
a) Resistance curves of the ZCO‐20 sensor in response to the volatiles emitted by 50 g of rice stored for different durations (15 consecutive responses). b) Variation of the sensor's response with storage time (left y‐axis) at room temperature, where the detection target is the gas released from the unhusked rice, along with the corresponding tetradecane release concentration (right y‐axis).

In this study, unhusked rice was further subjected to extended storage for 30 and 100 days under controlled conditions of 50 °C and 90% relative humidity. The concentration of released tetradecane was subsequently measured. As shown in **Figure** **8**a, the response value of the ZCO‐20 sensor increased from 1.2 to 16.9 with prolonged storage time, indicating high sensitivity to the odor associated with mold development. Furthermore, after 15 repeated measurement cycles on rice samples stored for different durations, the sensor consistently returned to its baseline resistance, demonstrating excellent repeatability. Figure 8b illustrates an excellent linear correlation (*R* ^2^ = 0.981) of the ZCO sensor's response during the first 100 days of monitoring. Based on the data in Figure S6 (Supporting Information), the theoretical tetradecane concentrations were estimated to be 1.96 ppm for fresh rice, 3.52 ppm after 30 days, and 6.22 ppm after 100 days of storage.^[^ ^61^ ^]^ Meanwhile, typical signs of early‐stage mildew, including darkened grain color (see inset in Figure 8b), were observed after 30 days of storage under constant temperature and humidity. Therefore, the sensor system proved capable of tracking the dynamic release of spoilage‐related VOCs during rice storage, indicating its potential for real‐time freshness evaluation and the prevention of mildew.

## 3 Conclusion 

This work presents the successful synthesis of microflower‐like Fe@WO_3_/ZCO heterostructures characterized by curved nanosheet‐like ZCO petals uniformly decorated with Fe@WO_3_ nanoclusters, using a one‐step solvothermal method. The careful regulation of Fe@WO_3_ loading within the range of 0 to 50 mg has been shown to enhance the sensitivity of the tetradecane gas sensor. Key attributes contributing to the superior sensing performance include a small crystallite size of 8.0 nm, high crystallinity of 77.2%, abundant oxygen vacancies, exposure on high‐energy (422) planes, and enhanced charge separation capabilities facilitated by the formation of multiple heterojunction interfaces. The n‐p junction sensor, specifically ZCO‐20, exhibits a low LoD of 78.4 ppb, whereas ZCO‐30 demonstrates the shortest t_rec_ of 36 s and achieves a response magnitude that is 6.8 times greater than that of pristine ZCO. ZCO‐30′s response to 80 ppm tetradecane was over 9.3 times higher than for other gases. Furthermore, ZCO‐30 exhibits excellent repeatability in its sensing performance. This work presents a robust and straightforward strategy for fabricating high‐performance, low‐power gas sensors that operate at room temperature based on heteroatom doping and p–n heterojunctions. These materials demonstrate strong potential as candidates for detecting large gas molecules. Importantly, this work also underscores the feasibility and prospects of applying MOS‐based sensors for the detection of long‐chain hydrocarbon gases.

## 4 Experimental Section

### Material Preparation

All chemicals used in the experiments were of analytical grade and were utilized without further processing. Sodium tungstate dihydrate (Na_2_WO_4_·2H₂O) (≥ 99.5%) and nitric acid (HNO_3_, 65–68 wt.%) were sourced from Sinopharm Chemical Reagent Co., Ltd. Anhydrous ferric chloride (FeCl_3_) (≥ 98%) was procured from Anhui Senrise Technology Co., Ltd. Anhydrous ethanol was obtained from Chinasun Specialty Products Co., Ltd. Cobalt nitrate hexahydrate (Co(NO_3_)_2_·6H_2_O), zinc nitrate hexahydrate (Zn(NO_3_)_2_·6H_2_O), and isopropanol ((CH_3_)_2_CHOH) were supplied by Chemical Reagents Company Limited of China Pharmaceutical Group.

Fe‐doped WO_3_ nanoparticles were obtained by a one‐step solvothermal method, as described in our earlier research.^[^ ^57^ ^]^ For this investigation, a concentration of 3% Fe‐WO_3‐x_ was selected. Initially, 10 mg of WO_3_@Fe nanoparticles were dispersed in 30 mL of isopropanol through ultrasonic treatment. Concurrently, 2 mmol of Zn(NO_3_)_2_·6H_2_O and 4 mmol of Co(NO_3_)_2_·6H_2_O were dispersed in isopropanol using ultrasonic agitation. Following this, 3 mL of absolute ethanol was gradually introduced into the solution while maintaining continuous stirring. The resulting mixture was then moved to a high‐temperature reactor and exposed to a temperature of 120 °C for 18 h. Following the reaction, the product was separated via centrifugation and then rinsed with deionized water and ethanol. The purified product was dried and subsequently annealed at 350 °C for 2 h, resulting in the formation of a 3D flower‐like Fe@WO_3_/ZnCo_2_O_4_ composite, as illustrated in Figure S1 (Supporting Information). The samples were categorized based on Fe@WO_3_ content (ranging from 0 to 50 mg) and were designated as ZCO, ZCO‐10, ZCO‐20, ZCO‐30, ZCO‐40, and ZCO‐50.

### Gas Sensor Fabrication and Measurements

The sensor electrodes were prepared on a 6 × 30 mm alumina substrate, with platinum paste screen‐printed to form the heater and measurement electrodes, maintaining a 0.42 mm gap for optimal design. Before applying the sensing film, the sensor substrates were thoroughly cleaned through sonication in deionized water, followed by rinsing with absolute alcohol. The coated sensors were preheated at 200 °C for approximately 10 h, followed by conditioning at room temperature.

Figure S2 (Supporting Information) shows a schematic of the gas sensing testing system. During the conditioning phase, high‐purity air (79% N_2_, 21% O_2_) continuously flowed over the sensors for a duration of 3 to 5 h to establish a stable baseline resistance. The tetradecane vapor pressure was 1 mmHg at 76.4 °C, with its concentration calculated using Equation (7)^[^ ^62^ ^]^ and regulated by a mass flow controller (MFC). An additional MFC was employed to control the flow of high‐purity blended air.

(7) $$\begin{array}{ccc} \mathrm{Concentration}\;\mathrm{of}\;\mathrm{VOCs}\;\left(\mathrm{ppm}\right) & = & \left(\mathrm{vapor}\;\mathrm{pressure}\;\mathrm{of}\;\mathrm{VOCs}\right.\\ & & \left.\left(\mathrm{mmHg}\right)/760\right)\times 10^{6} \end{array}$$

Data acquisition was conducted using a desktop computer with an analog‐to‐digital converter coupled with a laboratory DC power supply (GPS‐3303C) for the measurement of electrical resistance. The testing chamber was maintained at a controlled room temperature (25 ± 2 °C), with a relative humidity of approximately 15%–20%. Additional details regarding the gas sensor platform are available in our previous studies.^[^ ^41^ ^]^

The responses of the p‐type sensors were quantified as R_g_/R_a_, with R_a_ and R_g_ representing the equilibrium resistance in air and tetradecane. Response/recovery times (t_res_/t_rec_) refer to the time needed for resistance to achieve 90% of its total shift.

## Conflict of Interest

The authors declare no conflict of interest.

## Supporting information



Supporting Information

## Data Availability

The data that support the findings of this study are available from the corresponding author upon reasonable request.
