# Supplementary material for: Probing the Challenge of P‐Type Semiconductors in Long‐Chain VOC Detection: 3D Micro‐Flower Zinc Cobaltate Heterojunction Sensors
Source: Adv Sci (Weinh). 2025 Jun 20;12(34):e02646. doi: 10.1002/advs.202502646 (PMC12442688; doi:10.1002/advs.202502646)
Supplement: Supplementary file 1 — Supporting Information [file ADVS-12-e02646-s001.docx]

**Supporting Information**

**Probing the Challenge of P-Type Semiconductors in Long-Chain VOC Detection: 3D Micro-Flower Zinc Cobaltate Heterojunction Sensors**

*Kewei Liu^a, b ,c^, Zichen Zheng^a, b, d^**, Yiwen Zhou^a, b^, Carla Bittencourt^d^, Marc Debliquy^c^, Chao Zhang^a, b,^ **

a College of Mechanical Engineering, Yangzhou University, Yangzhou 225127, PR China

b Jiangsu Key Laboratory of Surface Strengthening and Functional Manufacturing, Yangzhou University, Yangzhou 225127, PR China

c Service de Science des Matériaux, Faculté Polytechnique, Université de Mons, Mons 7000, Belgium

d Research Institute for Materials Science and Engineering, Chimie des Interactions Plasma-Surface, University of Mons, 20 Place du Parc, Mons, 7000 Belgium

* Corresponding author

Prof. Chao Zhang

College of Mechanical Engineering

Yangzhou University

Huayang West Road 196

Yangzhou 225127, Jiangsu Province

P.R. China

**Material and Device Characterizations**

The crystal structure and elemental composition of the as-prepared samples were analyzed using an X-ray diffractometer (XRD) with Cu-Kα radiation (D8 Advance, Bruker, Germany). The microstructure and element distribution were confirmed using field-emission scanning electron microscopy (FESEM, S4800II, Hitachi, Japan) combined with energy-dispersive X-ray spectroscopy (EDS) and transmission electron microscopy (TEM, Tecnai G2 F30 S-TWIN, FEI, USA). The surface chemical state and oxygen vacancy analysis were studied through X-ray photoelectron spectroscopy (XPS, ESCALAB 250Xi, Thermo Fisher, USA). The UV absorbance was examined through a UV-vis-NIR spectrophotometer (Cary 5000, Varian, USA). FTIR spectra were recorded using the KBr pellet method on an infrared spectrophotometer with a resolution of 0.1 cm^-1^ in the range of 4000-400 cm^-1^. The specific surface areas with nitrogen adsorption-desorption at 77 K were measured using a Brunauer-Emmett-Teller (BET) instrument, and the pore diameter distribution of the materials was obtained through the Barret-Joyner-Halenda (BJH) method, utilizing the isotherms (Autosorb IQ3, Quantachrome Instruments, USA). Total pore volume ($\sum\Delta V_{p}$) is calculated from the desorption branch at P/P₀ = 0.99, converted to liquid volume using the density of liquid N_2_ (0.808 g/cm^3^). Pore size distributions were determined through complementary methodologies. During the desorption branch of a gas adsorption experiment, the actual pore radius $r_{p}$ corresponding to a specific relative pressure P/P_0_ is calculated by first determining the Kelvin radius $r_{k}$​, and then adding the statistical thickness t of the adsorbed layer present on the pore walls at that pressure (obtained from standard data or equations like Halsey's), resulting in $r_{p}$= $r_{k}$+t. The incremental gas volume desorbed at each pressure step, after correction for the thinning of the adsorbed film in larger pores already emptied, provides the pore volume $\Delta V_{p}$​ attributed to pores of radius $r_{p}$​. The volume-weighted average pore size is then calculated by summing the products of each $r_{p}$ and its corresponding $\Delta V_{p}$​, and dividing this sum by $\sum\Delta V_{p}$, effectively giving the mean pore radius weighted by the volumetric contribution of each pore size.

**DFT calculation details**

All density functional theory calculations were performed using the CASTEP code within Materials Studio. In the generalized gradient approximation (GGA), the exchange-related potential was described by the Perdew-Burke-Ernzerhof (PBE) functional. The 2×2×1 supercells of the (100) surface ZnCo_2_O_4_ were built. When performing geometric optimization, the energy cutoff of the plane wave expansion was set to 489.8 eV, and the SCF tolerance and k-point were set to fine. A 15 Å vacuum layer was set between the layers to avoid interactions. The diffraction peaks of the XRD pattern demonstrated that the synthesized ZnCo_2_O_4_ corresponded well to the cubic structure (Fd-3m (227) space group).


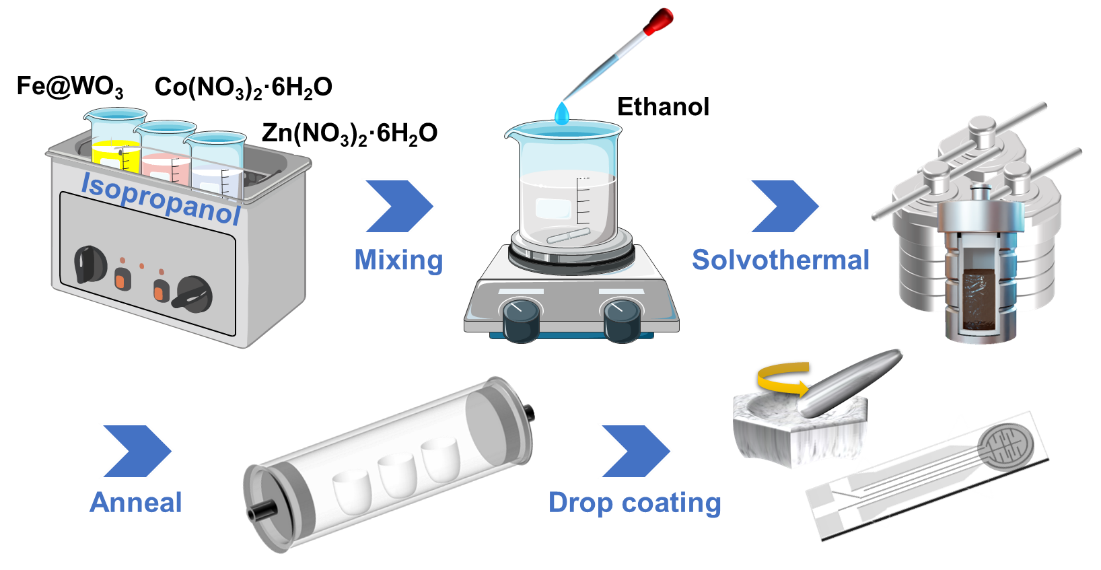


**Fig. S1** Schematic images of the synthesis of Fe@WO_3_/ZnCo_2_O_4_ composite.


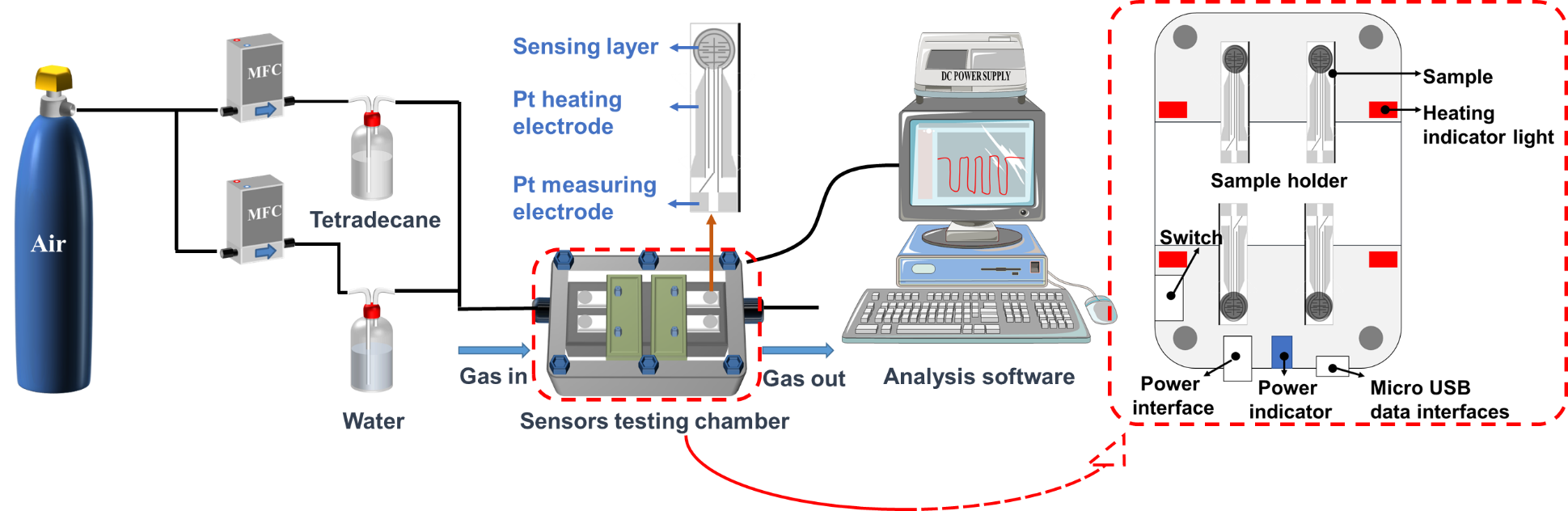


**Fig. S2** Schematic diagram of the gas sensing testing system.

**
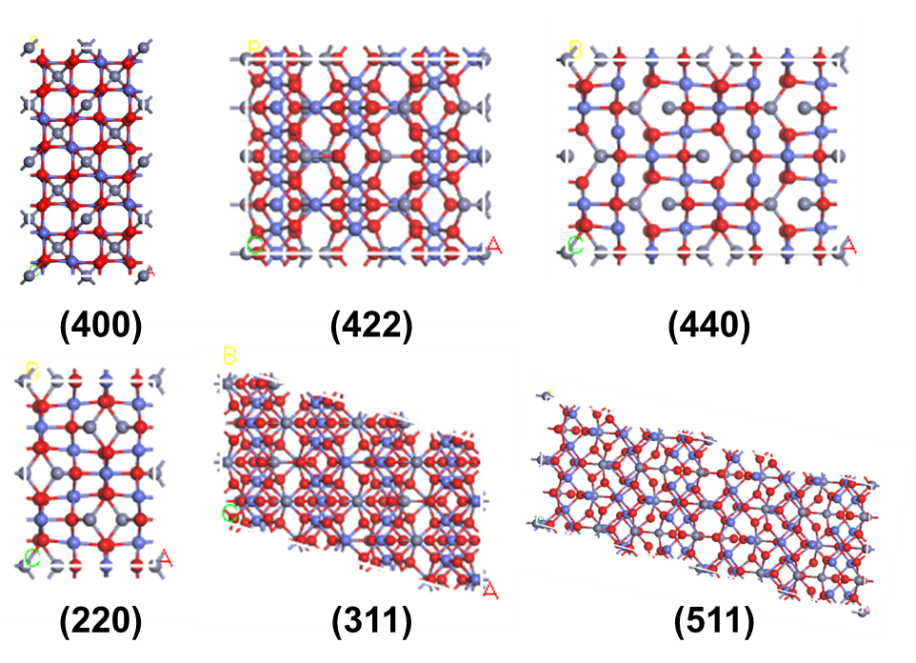
**

**Fig. S3** Optimized surface structures of ZnCo_2_O_4_ (hkl) facets based on DFT calculations

**
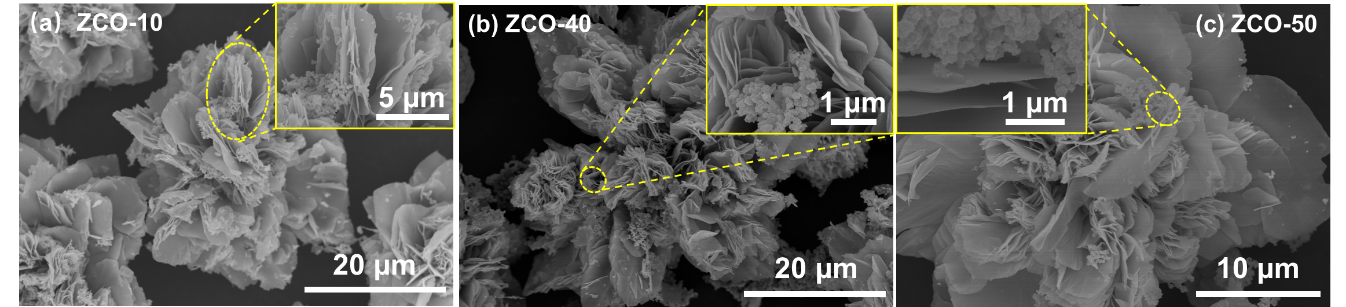
**

**Fig. S4** FE-SEM images of (a) ZCO-10, (b) ZCO-40 and (c) ZCO-50. Inset: the magnified SEM images of them.

**
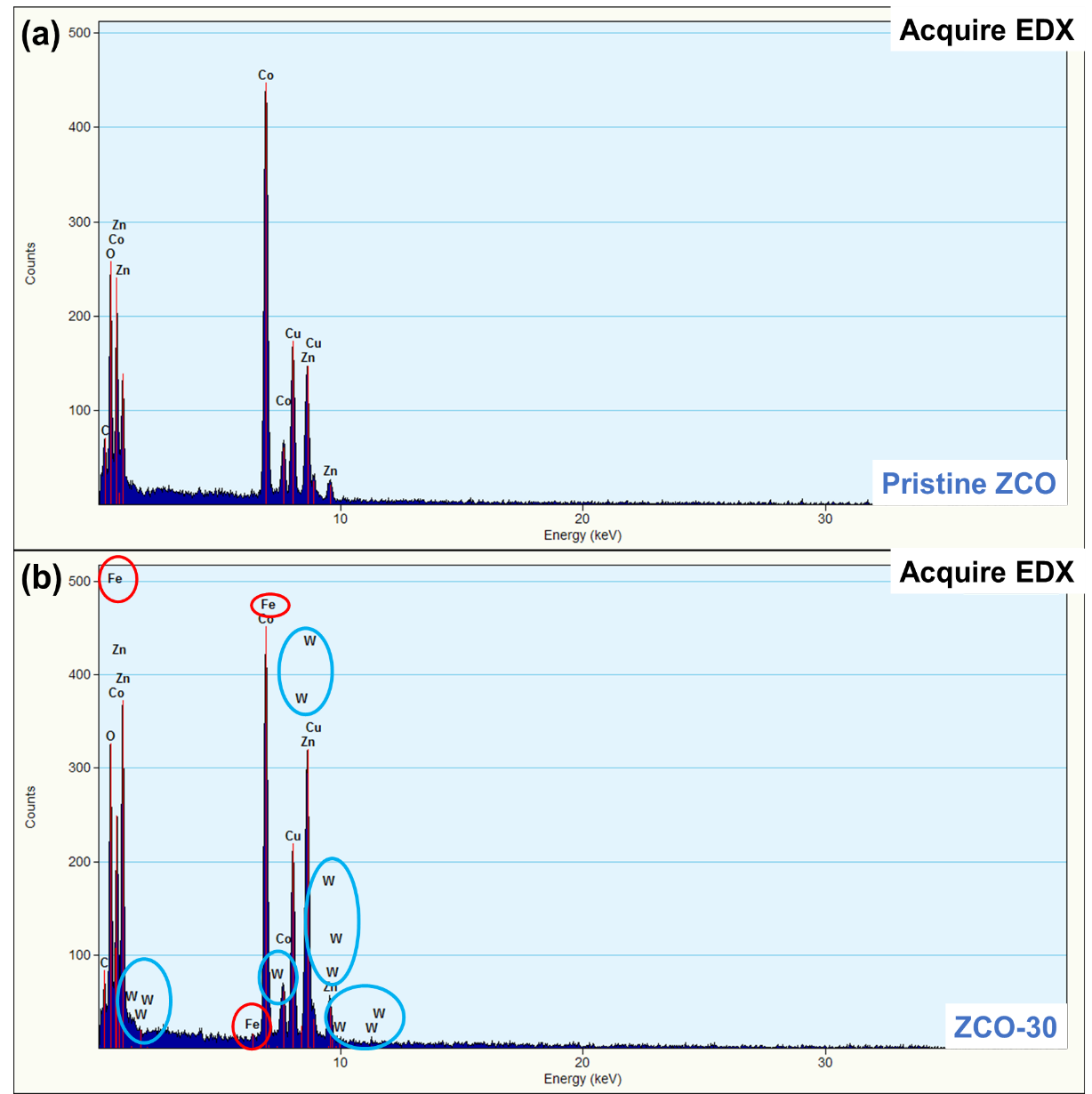
**

**Fig. S5** EDX spectra of ZCO and Fe@WO_3_/ZnCo_2_O_4_ heterostructure. The red circles represent the Fe element, and the blue circles represent the W element.

**Fig. S6** Dual-logarithm of response and tetradecane concentration of 60-300 ppm for ZCO-20. Inset: 100 consecutive data points were taken during reference resistance stabilization for calculating the sensor standard deviation.


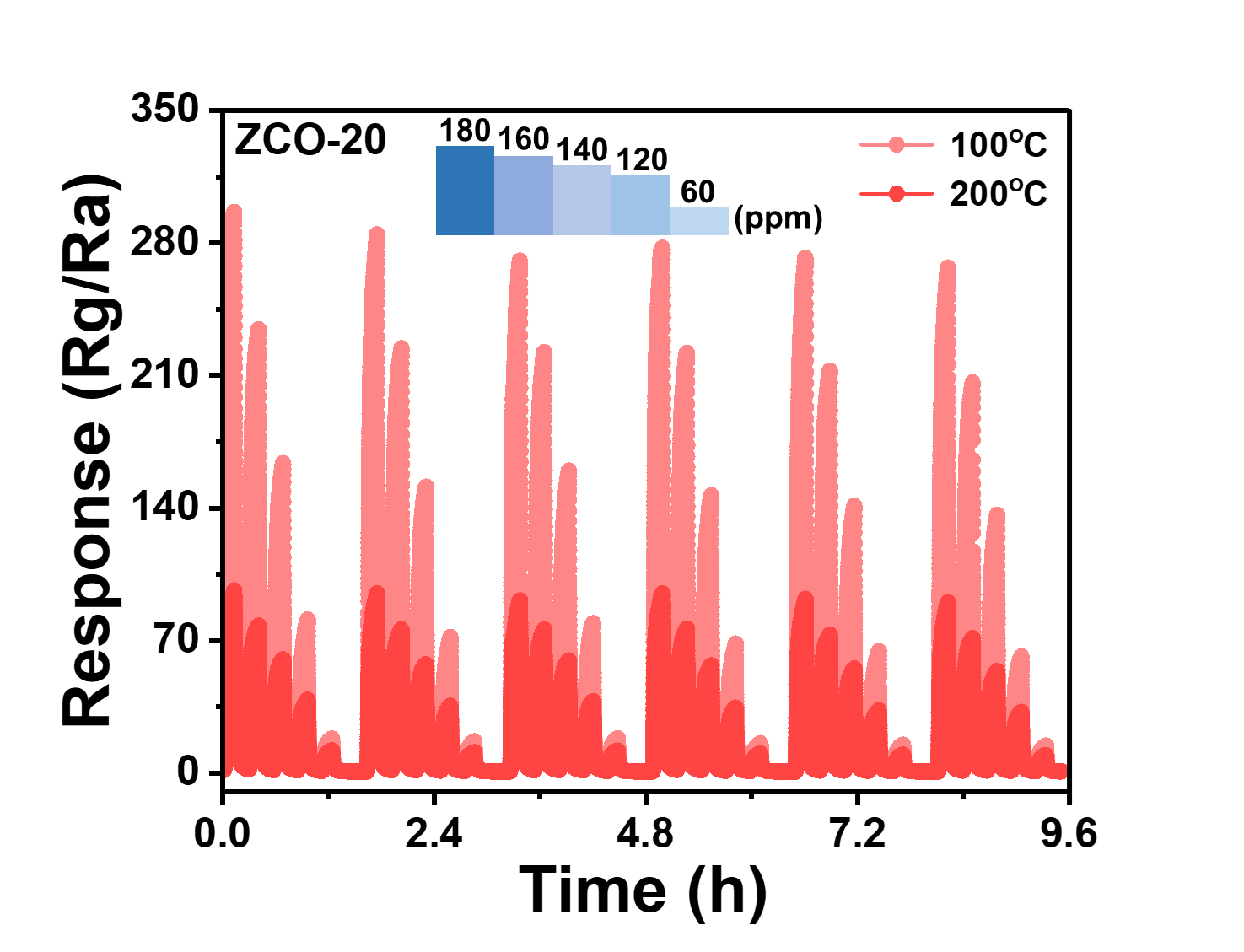


**Fig. S7** Dynamic response curves of the ZCO-20 sensor toward 180–60 ppm tetradecane at operating temperatures of 100^o^C and 200^o^C.


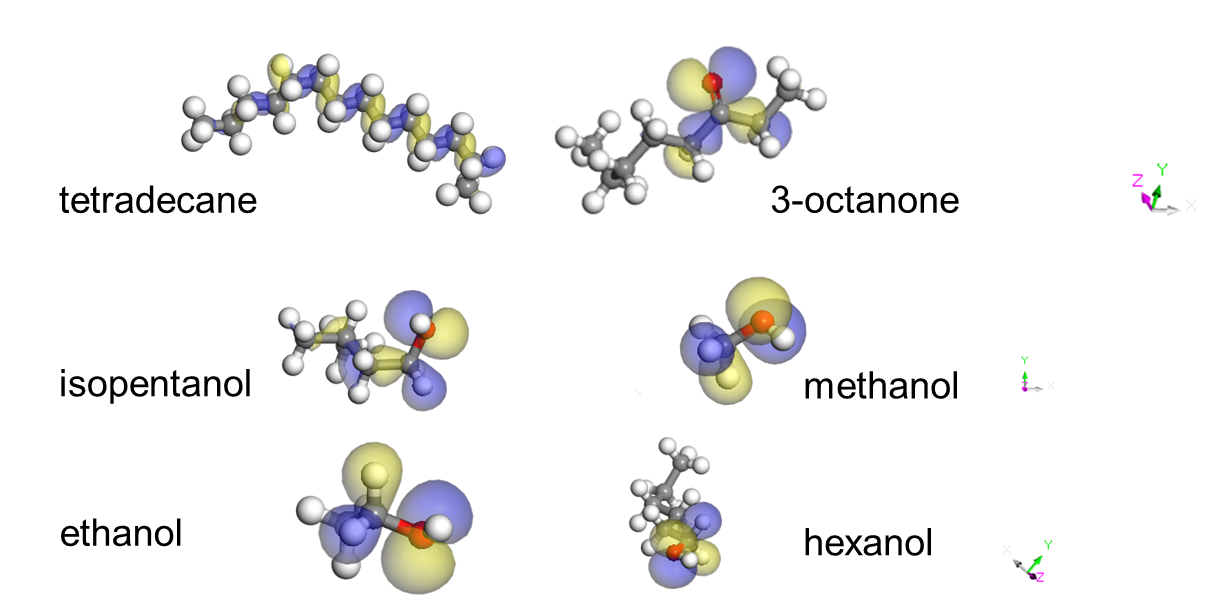


**Fig. S8** Visualization of the highest occupied molecular orbital (HOMO) of the target gas molecules.

**
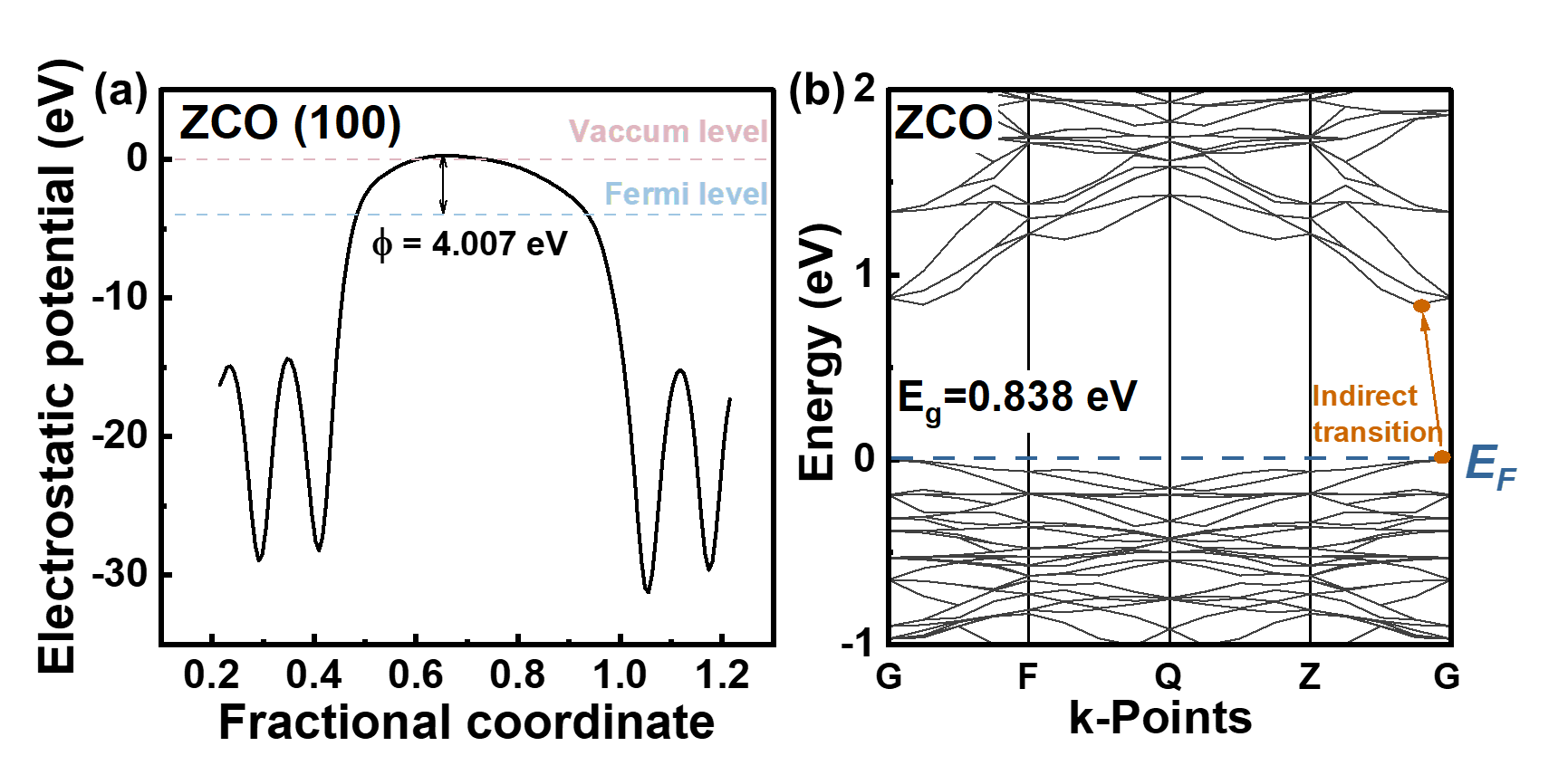
**

**Fig. S9** (a) Band structure of ZCO. (b) Electrostatic potentials of ZCO (100).

**Fig. S10** Schemic adsorption configuration for MD.


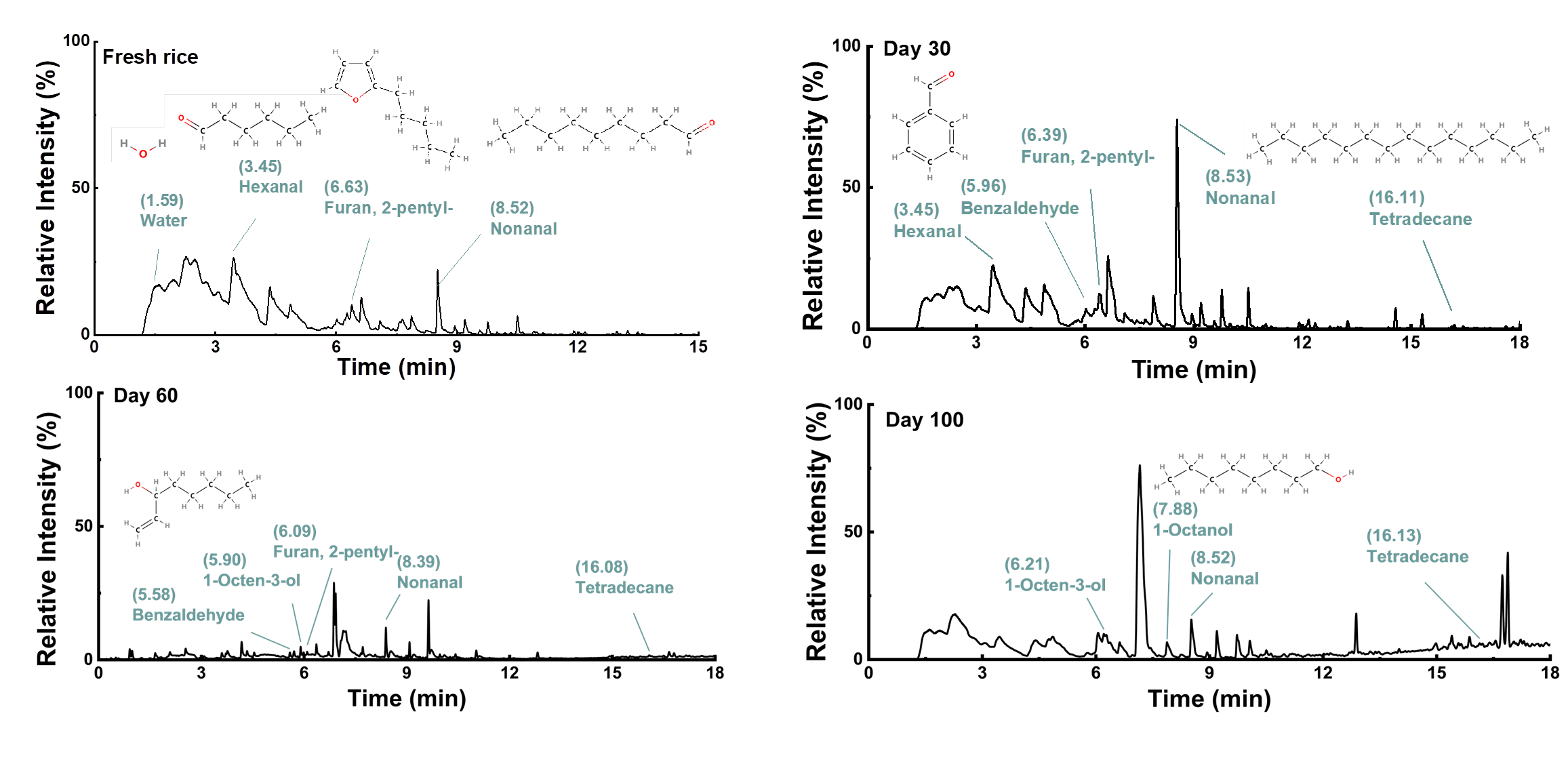


**Fig. S11** VOCs profiling of unhusk rice samples stored at different times obtained by GC-MS.

**Table S1** Physical properties of all samples calculated via XRD results.

| **Sample** | **Average grain size**  **D (nm)** | **Crystallinity**  **(%)** | **Micro strain**  **ε*10^−3^** | **Dislocation density**  **δ*****10^−3^ (nm^-2^)** |
| --- | --- | --- | --- | --- |
| **ZCO** | 8.677 | 74.86 | 13.706 | 10.451 |
| **ZCO-10** | 8.904 | 64.03 | 12.905 | 10.010 |
| **ZCO-20** | 9.230 | 81.49 | 12.186 | 9.745 |
| **ZCO-30** | 8.015 | 77.16 | 15.918 | 11.107 |
| **ZCO-40** | 8.869 | 73.38 | 12.999 | 10.075 |
| **ZCO-50** | 8.637 | 73.95 | 13.656 | 10.317 |

**Table S2** TC values and standard deviation of all samples.

| **Sample** | **TC_(220)_** | **TC_(311)_** | **TC_(400)_** | **TC_(422)_** | **TC_(511)_** | **TC_(440)_** | **σ** |
| --- | --- | --- | --- | --- | --- | --- | --- |
| **ZCO** | 1.323 | 0.802 | 1.324 | 1.138 | 0.679 | 0.735 | 21.857 |
| **ZCO-10** | 1.046 | 0.907 | 1.282 | 1.112 | 0.755 | 0.888 | 23.239 |
| **ZCO-20** | 1.124 | 0.834 | 1.265 | 1.355 | 0.705 | 0.717 | 24.093 |
| **ZCO-30** | 1.083 | 0.944 | 1.231 | 1.145 | 0.777 | 0.820 | 26.180 |
| **ZCO-40** | 1.109 | 0.942 | 1.180 | 1.094 | 0.804 | 0.871 | 25.743 |
| **ZCO-50** | 1.102 | 0.862 | 1.147 | 1.296 | 0.728 | 0.864 | 24.968 |

**Table S3** Calculated HOMO, LUMO energy levels, and energy gaps (ΔE) of selected VOCs using the DMol3 module.

| **Analyte** | **HOMO** | **LUMO** |
| --- | --- | --- |
| **tetradecane** | -0.248568 | 0.064838 |
| **methanol** | -0.218027 | 0.040603 |
| **hexanol** | -0.21289 | 0.043668 |
| **ethanol** | -0.214637 | 0.041825 |
| **3-octanone** | -0.193291 | -0.044211 |
| **isopentanol** | -0.214202 | 0.037794 |

HOMO and LUMO energy levels were calculated using the DMol3 module based on DFT without vacuum level alignment.
